# Prise en charge hospitalière de la malnutrition aigue sévère chez l'enfant avec des préparations locales alternatives aux F-75 et F-100: résultats et défis

**DOI:** 10.11604/pamj.2015.21.329.6632

**Published:** 2015-08-31

**Authors:** Félicitée Nguefack, Chritoph Akazong Adjahoung, Basile Keugoung, Nelly Kamgaing, Roger Dongmo

**Affiliations:** 1 Faculté de Médecine et des Sciences Biomédicales, Université de Yaoundé I, Cameroun; 2 Groupe Associatif pour la Recherche, l'Education et la Santé (GARES-Falaise), Dschang, Cameroun; 3 Hôpital Général de Douala, Cameroun; 4 Centre Hospitalier Universitaire de Yaoundé, Cameroun; 5 Hôpital de District d'Efoulan, Yaoundé,Cameroun

**Keywords:** Malnutrition sévère, laits thérapeutiques F75/F100, ingrédients locaux, enfants, severe malnutrition, therapeutic milks F75/F100, local ingredients, children

## Abstract

**Introduction:**

La mise en œuvre des directives de l'OMS permettrait de réduire significativement la mortalité hospitalière due à la malnutrition sévère. Cependant, elle n'est pas effective et la pénurie en aliments thérapeutiques est l'une des principales causes. L’étude décrit notre expérience sur la prise en charge hospitalière de la malnutrition aigue sévère avec des laits alternatifs aux F75 et F100 composés localement.

**Méthodes:**

Il s'agissait d'un essai clinique non randomisé. La prise en charge des patients utilisait les laits composés localement et une évaluation quotidienne du gain pondéral était faite.

**Résultats:**

L’étude a porté sur 41 sujets âgés de 6 à 59 mois. Au total, 73,2% avaient le kwashiorkor-marasmique, 17,0% le kwashiorkor, 9,8% le marasme et 41,5% étaient infectés par le VIH. Nous avons noté une prise progressive du poids d'environ 10 g/kg/jour vers le 7ème jour et de 15 à 20 g/kg/jour en fin d'hospitalisation. Le taux de mortalité était de 21,9% soit une réduction de 8,4% des chiffres antérieurs.

**Conclusion:**

Malgré les obstacles financiers liés au coût des ingrédients, les préparations lactées alternatives aux standards F75 et F100, sont adaptables dans notre contexte. En l'absence des formules standards de l'OMS et lorsque la référence vers une structure qui en disposent n'est pas possible, les préparations locales permettraient de réhabiliter efficacement les patients. D'autres recherches pointues permettraient de tirer les ingrédients uniquement de notre environnement. Elles contribueraient ainsi à minimiser les couts des préparations et de favoriser la pérennisation des laits thérapeutiques locaux.

## Introduction

La mortalité infantile était très importante avant les années 1990 et dépassait 30% chez les enfants souffrant d'une malnutrition sévère [1]. Au Cameroun, le protocole de prise en charge de la malnutrition aigue sévère élaboré par le Ministère de la santé repose sur les nouvelles directives de l'OMS. L'application rigoureuse de celles-ci a permis de réduire la mortalité infantile dans d'autres contextes [1–3]. Cependant, leur mise en œuvre n'est pas toujours effective à tous les niveaux. En effet, il y a une pénurie en intrants notamment les solutions de réhabilitation F75 et F100 dans la plupart des formations sanitaires. Seuls deux hôpitaux tertiaires de Yaoundé reçoivent des aliments thérapeutiques. La plupart des sites ne sont pas capables de mettre en œuvre les recommandations de l'OMS en matière de prise en charge de la malnutrition sévère. De plus, l'organisation du système de transfert, de référence et de contre référence entre les échelons adéquats de prise en charge n'est pas encore structurée dans la plupart des districts de santé du Cameroun. Comme ailleurs, la stratégie de renforcement continu des compétences y compris dans la prise en charge de la malnutrition sévère n'est pas toujours opérationnelle [4]. Dans notre site, on utilisait jusque là des bouillies dites enrichies pour la réhabilitation des sujets souffrant de la malnutrition sévère. Des préparations alternatives aux bouillies de céréales et aux préparations de l'OMS ont été produites et utilisées afin de palier à l'absence des ces dernières. L'objectif de cette étude est de décrire notre expérience sur l'utilisation des laits obtenus à partir des produits disponibles localement pour la prise en charge de la malnutrition sévère chez l'enfant et d’évaluer son impact sur la prise pondérale.

## Méthodes

Un essai clinique non randomisé portait sur 41 enfants hospitalisés pour malnutrition aigue sévère. Elle a eu lieu dans le service de nutrition du Centre Mère et Enfant, une structure hospitalière pédiatrique de Yaoundé. Ce centre reçoit annuellement entre 7000-9000 hospitalisations parmi lesquelles 230-265 patients malnutris.

**Population**: nous avons enrôlé tous les enfants âgés de 6 à 59 mois admis entre le 1er avril et le 30 septembre 2007 pour une malnutrition sévère. Elle est évoquée chez un enfant lorsqu'il a soit des œdèmes nutritionnels bilatéraux des membres inférieurs ou un indice anthropométrique Poids/Taille (P/T) inférieur à -3 Z-score selon les valeurs médianes de référence de l'OMS [5]; ou encore lorsque son périmètre brachial (PB) est inférieur à 115 mm.

Préparation des laits thérapeutiques et étapes de prise en charge des patients:des laits nutritifs alternatifs au F75 et F100 de haute valeur énergétique ont été reconstitués à partir des produits disponibles sur le marché local (pharmacies ou supermarchés par exemple). Il s'agit d'un lait entier (Nido^®^), de l'huile de table, du sucre en carreau, des céréales (Cérélac^®^), des multivitamines et des minéraux fournis par le complexe Gènes vit plus^®^ auquel nous ajoutions des Oligo essentiels Zinc^®^ et Oligo essentiels Cuivre^®^ ainsi que du KCl à 10% (Tableau 1). L’étude a bénéficié de l'appui du site et de l’équipe de recherche. Le personnel a été formé à la préparation des laits enrichis et son administration selon les principes de base tels que recommandés par l'OMS [6]. La prise en charge de la malnutrition sévère comportait deux phases dont une initiale de stabilisation et une deuxième de réhabilitation nutritionnelle. Dans la première phase, les complications étaient traitées et on apportait à chaque sujet 80 à 100 kcal/kg/jour, 1-1,2 gr/kg/jour de protéines avec le lait F75 local correspondant à environ 75 kcal/100 ml. Afin de corriger d'autres carences concomitantes, les patients recevaient une dose unique de 5 mg d'acide folique et de vitamine A à raison de 50000, 100000 ou 200000 UI selon qu'ils étaient âgés de moins de 6 mois, 6-12 ou de plus 12 mois respectivement. La deuxième phase débutait lorsque le patient remplissait les critères requis après une transition conditionnée par un retour de l´appétit. Les mères des patients encore allaités, étaient encouragées à poursuivre l'allaitement. La suite de la réalimentation se faisait avec un lait alternatif au F100 qui apportait environ 130 Kcal/kg/jour soit 100 kcal/100 ml et la même quantité de supplément vitaminique et minéral que pour le F75. Les apports énergétiques pouvaient être augmentés par paliers jusqu’à 200 kcal/kg/jour en introduisant des bouillies habituellement fournies dans le site et progressivement, les repas solides comme recommandé par des auteurs [7]. Cette augmentation tenait compte de la demande du patient surtout de ceux qui consommaient toute leur ration plus tôt et constituait aussi une étape de préparation à la sortie de l'hôpital. Le fer était administré à raison de 2 mg/kg/jour à cette phase. Par ailleurs, les patients étaient soumis au traitement antibiotique empirique [2]. La sortie était envisagée lorsque le poids pour l’âge avoisinait 85% en l'absence d’œdèmes avec une reprise de la vigueur et aussi lorsque les parents étaient conseillés par rapport à la poursuite de l'alimentation équilibrée de l'enfant avec les produits locaux [8].

**Tableau 1:** Composition des préparations alternatives adaptées localement F75 et F100

Produits utilisés	Quantité pour 1 litre de solution alternative au F75*		Quantité pour 1 litre de solution alternative au F100**	
Lait entier en poudre	35 g	7 cuillères mesures	110 g	21 cuillères mesures
Sucre de commerce	70 g	14 carreaux	50 g	10 carreaux
Huile de soja	20 g	23 ml	30 g	35 ml
Farine de riz ou autre céréale cuite	35 g	7 cuillères mesures		
Supplément vitaminique et minéral	3,2 g	1 comprimé de multivitamine *** + Oligo éléments**** + 22,5 ml de KCl à 10%	3,2 g	1 comprimé de multivitamine*** + Oligo éléments**** + 22,5 ml de KCl à 10%

Mélange porté à 1 litre avec de l'eau potable

* 1 litre de la solution correspond à 76,1 Kcal;

** 1 litre de la solution locale F100 correspond à 97,6 Kcal ;

*** Multivitamines (Gènes vit Plus^^®^^) ;

**** Oligo-éléments constitués de 1,5 ml d'Oligo essentiel Zinc + 5 ml d'Oligo essentiel Cuivre)

**Anthropométrie**: le poids était mesuré dès l'admission et tous les jours à l'aide d'une balance électronique mère/enfant de marque SECA dont la portée est de 150 Kg avec une précision de ± 100 g. La mesure de la taille se faisait grâce à un enfantomètre du modèle Unicef fabriqué localement et le périmètre brachial (PB) avec un mètre de type Unicef. Les œdèmes étaient recherchés aux deux membres inférieurs. Leur présence ou pas, ainsi que les indices anthropométriques et le PB permettaient de classer et de suivre l’évolution des sujets en cours d'hospitalisation. Le poids minimum d'un enfant atteint de kwashiorkor correspondait à celui observé après la fonte totale des œdèmes. Nous avons recherché les pathologies chroniques et décrit les aspects démographiques, cliniques ainsi que l’évolution en cours d'hospitalisation.

**Analyses statistiques**: les données étaient saisies et analysées à l'aide des logiciels Excel et Epi-info. L’évolution du statut nutritionnel était évaluée par le gain pondéral et par l'indice anthropométrique Poids/Taille. Ce dernier était calculé à l'aide du logiciel WHO Anthro version 3.2; 2005 et exprimé en Z-score. Le gain pondéral en g/kg/jour était estimé quotidiennement par la formule ((Poids observé - poids initial)/(Poids moyen x nombre de jours d'hospitalisation)) x 1000 [9]. Nous avons traduit les variables catégorielles en pourcentage, tandis que les variables continues étaient exprimées en médianes, moyennes et écart type. La différence entre les proportions était significative pour les valeurs de p< 0,05.

**Ethique de la recherche**: l’étude a été approuvée par le comité institutionnel d’éthique de la Faculté de Médecine et des sciences Biomédicales de l'Université de Yaoundé I. L'approbation des parents était obtenue préalablement après un consentement écrit ou verbal. Aucun acte invasif n’était réalisé, et les parents étaient libres de refuser sans aucune influence sur le suivi de leurs enfants. Aucun parent n'a refusé de participer à l’étude et ne s'est retiré en cours d’étude.

## Résultats

**Données sociodémographiques**: au total, 41 enfants hospitalisés pour malnutrition aigue sévère étaient pris en charge durant la période d’étude. Leur âge médian était de 17 mois (intervalle interquartile de 13-22 mois et extrêmes de 8 à 34 mois). La tranche d’âge de 12 à 23 mois était la plus représentée (Tableau 1). Les garçons étaient plus nombreux (24 soit 59%) contre 17 filles (41%). La plupart des mères (83,0%) étaient agées de 15 à 24 ans, avec un âge médian de 20 ans (intervalle interquartile 19-23 ans). Elles vivaient majoritairement seules (68,3%) et de façon générale, le revenu mensuel des familles était inférieur à 50000 FCFA (Tableau 2).

**Tableau 2:** Données sociodémographiques

		Effectif (%)
Age des enfants (mois)	6 – 11	9(21,9)
	12 – 23	25(60,0)
	24 – 35	7 (17,1)
Age des mères (années)	15 – 19	15(36,6)
	20 – 24	19(46,4)
	25 – 35	6(14,6)
	≥ 36	1(2,4)
Niveau d'instruction des mères	Non scolarisée	4(9,8)
	Primaire	7(17,0)
	Secondaire	30(73,2)
Statut matrimonial des mères	Vie en couple	13(31,7)
	Non en couple	28(68,3)
Revenu mensuel (FCFA)	< 50 000	33(80,5)
	50000 - 100000	8(19,5)

**Figure 1:**
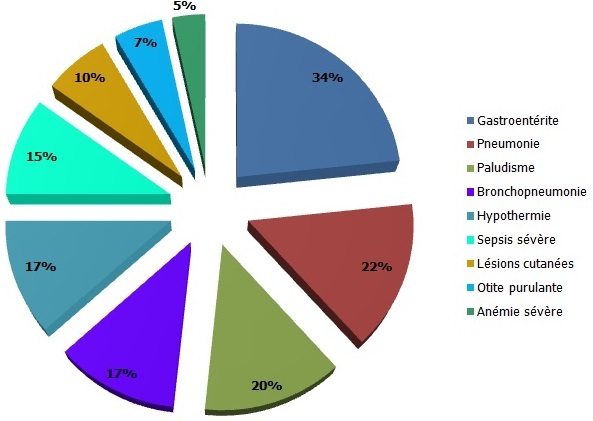
Fréquence des complications retrouvées chez les patients

**Données cliniques**: près de 3/4 des patients (73,2%) avaient le kwashiorkor-marasmique, 17,0% le kwashiorkor et 9,8% le marasme. Le PB moyen était de 99,8 mm à l'admission et 43,9% de sujets étaient en malnutrition profonde avec un P/T< - 5 et ≤ - 4 chez 24,3% et 19,6% respectivement. Par ailleurs, 41,5% étaient infectés par le VIH et il y avait 7,3% de drépanocytaires. Les complications les plus fréquentes à l'admission étaient infectieuses et présentes chez plus de 70% des sujets (Figure 1). La gastroentérite dominait le tableau (34%) puis suivaient les infections respiratoires basses notamment la pneumonie et la bronchopneumonie (31%). Tous les cas de sepsis sévère se retrouvaient chez les sujets infectés par le VIH.

**Figure 2:**
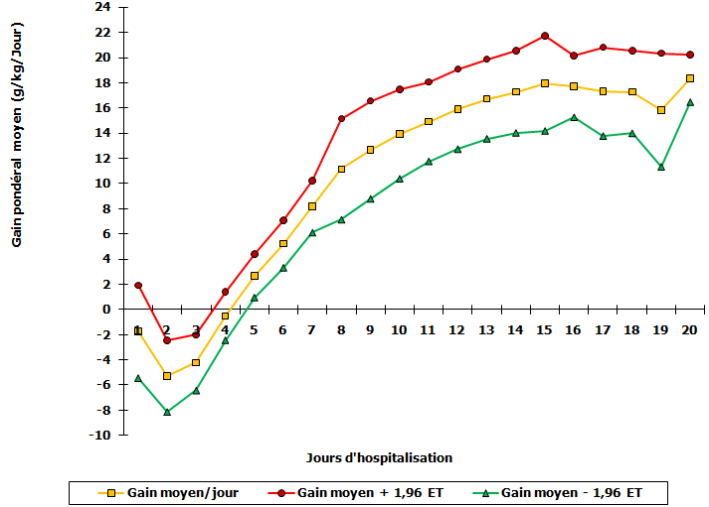
Evolution quotidienne du gain pondéral moyen en cours d'hospitalisation

**Evolution sous réhabilitation nutritionnelle**: nous avons observé chez certains sujets une perte de poids pendant les 48 premières heures et une reprise progressive jusqu´à 10 g/kg/jour au 7ème jour, puis de 15 à 20 g/kg/jour à la sortie (Figure 2). La durée d'hospitalisation allait de 1 à 21 jours, avec une médiane de 17,5 jours (intervalle interquartile 12-18 jours). Chez l'un des patients, la phase de stabilisation était très longue de 9 jours, avec une stagnation et une baisse du poids, d´où la cassure de la courbe au 20e jour (Figure 2). La durée de la phase de stabilisation était plus courte (moins de 4 jours) chez tous les patients marasmiques; elle était plus élevée en cas de kwashiorkor avec 85,7% au delà de 5 jours, mais la différence n’était pas significative (P= 0,0819). Quant au kwashiokor-marasme, près de la moitié des patients (51,7%) étaient stables au bout des 4 premiers jours; 44,8% l’étaient entre 5 et 7 jours alors qu'un seul s’était stabilisé au-delà de cette période. Les patients qui souffraient d'une gastroentérite associée au paludisme n’étaient stables cliniquement qu'entre 5 et 7 jours voire plus, contrairement aux autres qui l’étaient un plus tôt (P= 0,0002). La durée d'hospitalisation variait de quelques heures à 21 jours avec une moyenne de 13,3 + 6,5 jours. Il n'y avait pas de différence significative entre ce paramètre et le type de malnutrition. La plupart des patients séjournaient entre 15 et 19 jours notamment ceux qui avaient la gastroentérite associée au paludisme ainsi que ceux qui souffraient de la pneumonie. Par contre, ceux qui avaient le sepsis sévère séjournaient moins de 9 jours (p= 0,0010). L’évolution était favorable chez 31 patients (75,6%) contre neuf décédés soit une mortalité spécifique de 21,9%. Tous les décès étaient enregistrés dans les 72 heures d'admission avec plus de 3/4 (78%) pendant les 48 premières. Presque tous 8/9 (89,0%) survenaient chez les patients qui avaient le kwashiorkor-marasmique contre un décès (11,0%) chez les marasmiques. Près de 2/3 (67,0%) et le tiers (33,0%) des décédés avaient un sepsis sévère et une pneumonie massive respectivement. Tous les décès survenaient chez les patients infectés par le VIH. Au total, 78% des sujets avaient atteint 85% du poids cible pour l’âge, seuil requis pour la sortie. En dehors d'un patient, qui avait maintenu un indice P/T <-3 Zscore, la majorité (59,4%) était autour de la médiane (Tableau 3).

**Tableau 3:** Données cliniques

		Effectif (%)
Type de malnutrition n =41	Marasme-kwashiokor	30 (73,2)
	Kwashiokor	7 (17,0)
	Marasme	4 (9,8)
Z-Score Poids/Taille à l'admission n =41	< - 5	10 (24,3)
	≤ - 4	8 (19,6)
	< - 3	23 (56,1)
Délai de reprise totale de l'appétit (jours) n =41	≤ 6	6 (24,4)
	7 – 12	3 (4,9)
	13 – 18	17 (51,2)
	19 – 21	8 (19,5)
Durée de la phase de stabilisation (jours) n =32	1 – 4	11 (34,4)
	5 – 6	19 (59,4)
	8 – 10	2 (6,2)
Durée de la phase de réhabilitation (jours) n =32	7 – 10	9 (28,1)
	11 – 16	23 (71,9)
Infecté par le VIH n = 41	Oui	17 (41,5)
	Non	14 (34,1)
	Statut inconnu	10 (24,4)
Z Score Poids/Taille à la sortie	< -3	1 (3,1)
	-2 à -1	10 (31,3)
	-1 à +1	19 (59,3)
	≥ +2	2 (6,3)
Statut VIH des décédés N = 9	Positif	9 (100)
	Négatif	0 (0)
Age des décédés (mois) N = 9	< 12	3 (33,3)
	12 – 23	4 (44,5)
	≥ 24	2 (22,2)
Délai de survenue des décès (jours)	≤ 2	6 (66,7)
	3 - 4	3 (33,3)

## Discussion

**Accessibilité aux laits thérapeutiques localement adaptés**: les laits thérapeutiques adaptés localement constituent une alternative intéressante pour la réhabilitation des patients souffrant d'une malnutrition sévère dans un contexte à ressources limitées. Toutefois, leur coût élevé reste une barrière à surmonter. En effet, les ingrédients nécessaires pour la préparation exigent près de 13 $US au démarrage, soit environ 4 et 4,5 $US par litre de solution de 75 et F100 respectivement. Ces préparations peuvent couter jusqu’à 40 $US selon la durée de la récupération nutritionnelle. Elles ont aussi par ailleurs la particularité d’être contraignantes en temps alloué et nécessitent un réfrigérateur pour une conservation d'au plus 24 heures et un usage exclusivement hospitalier. Ce n'est pas le cas pour d'autres aliments thérapeutiques adaptés localement qui peuvent être utilisés dans les communautés [10, 11]. En l'absence de subvention, il serait difficile de pérenniser ces aliments dont les ingrédients sont achetés et dont les obstacles financiers réduiraient leur utilisation dans toutes les communautés. En Ethiopie, les coûts d'opportunité constituaient une barrière à l'utilisation des services de traitement de la malnutrition sévère [12]. En Ouganda, une formule permettant de préparer des bouillies locales à moindre coût a été développée pour la réhabilitation nutritionnelle; leur implémentation a permis de diminuer la mortalité de 14,2% ainsi que les échecs thérapeutiques [13]. Des recherches plus approfondies permettraient d'obtenir des formules avec des ingrédients tirés uniquement de notre environnement afin de minimiser les coûts des laits thérapeutiques.

**Mise en œuvre des directives de prise en charge de la malnutrition aigue sévère selon l'OMS**: la prise en charge adéquate de la malnutrition nécessite un personnel qualifié et un système de santé performant qui permet de sauver un maximum de vie [14]. Alors que dans certains contextes les auteurs réfléchissent sur les moyens d'avoir un personnel compétent [4], notre milieu fait face en plus à l'indisponibilité des aliments standards. Même avec les ressources adéquates disponibles, certains sollicitent des efforts supplémentaires pour améliorer convenablement la survie des patients souffrant de la malnutrition sévère [15]. La composition des laits nutritifs dans notre site a été le point de départ pour l'amélioration de la prise en charge de la malnutrition dans certains hôpitaux de Yaoundé qui ne disposent pas des solutions standards de l'OMS.

**Evolution des patients pendant l'hospitalisation**: le poids cible était rapidement atteint chez certains patients avec un gain atteignant 15g/kg/jour en moyenne à la fin de l'hospitalisation. La prise pondérale était précédée d'une baisse durant les 72 premières heures et correspondait à la fonte des œdèmes chez ceux qui avaient la malnutrition œdémateuse. Cette évolution pondérale était superposable à celle qui était notée par certains auteurs lorsque le réhabilitation nutritionnelle se faisait avec la solution standard F75 et F100 [7]. Par contre, le gain pondéral était moins élevé chez les patients dans une étude réalisée dans un centre de réhabilitation utilisant les solutions standards en Inde [15]. Dans la présente étude, tous les patients n'ont cependant pas atteint le poids cible avant la sortie. En Colombie, 61,7% des patients avaient atteint un indice P/T de -1 z-score, 3 semaines après le début de la réhabilitation nutritionnelle [16]. Ceci n'est pas toujours possible en peu de temps d'observation dans un contexte comme le notre où certains patients avaient des comorbidités, à l'instar de l'infection à VIH. Des auteurs ont démontré que la vitesse de récupération dépendait du degré de déficit nutritionnel initial [17]. Près de la moitié des sujets avaient une malnutrition très profonde à l'admission. Cette situation n'aurait pas été en faveur d'une récupération nutritionnelle appréciable. Par ailleurs, l'infection à VIH contribuerait à la retarder également [18]. A Kumasi, elle était associée à un gain de poids faible et une mortalité élevée [19]. Selon la même étude, l'infection à VIH était retrouvée chez 27,2% des patients [19]. L'effet du VIH sur la croissance des enfants et l'inversion de celui-ci par le traitement antirétroviral sont prouvés [20]. Dans notre étude, 41,5% des sujets étaient infectés par le VIH et ils n’étaient pas sous traitement antirétroviral. L'implémentation des directives de l'OMS permettrait de réduire considérablement la mortalité dans la malnutrition sévère [16]. Notre intervention a contribué à diminuer de 8,3% la mortalité par rapport aux taux qui étaient enregistrés lorsque la prise en charge se faisait avec les bouillies dont les valeurs protéiques, caloriques et minérales étaient peu maitrisées. Des auteurs ont rapporté des résultats similaires en Afrique du Sud et au Ghana [3]. Les sujets avec kwashiorkor-marasmique prédominaient (73%) dans notre étude et c'est également dans ce groupe que les décès survenaient. Ces décès survenaient en début d'hospitalisation et s'observaient essentiellement chez les patients infectés par le VIH. Le même constat a été fait au Malawi, les sujets souffrant d'une malnutrition aigue sévère mouraient plus que les autres lorsqu'ils étaient infectés par les VIH [21]. En dehors de ce contexte, les décès survenaient beaucoup plus chez patients dont les indices anthropométriques étaient très affectés [22]. Par ailleurs, le succès thérapeutique dépend de la qualité de la prise en charge des complications [23].

## Conclusion

Malgré les coûts élevés des ingrédients, les préparations lactées alternatives aux standards F75 et F100 sont adaptables dans notre contexte et permettraient de réhabiliter efficacement les patients lorsque l'approvisionnement en laits standards ou que la référence vers d'autres structures de prise en charge s'avère impossible. Associées au traitement empirique, ces préparations alimentaires pourraient réduire le taux de mortalité qui reste préoccupant et exige une amélioration générale de la qualité de prise en charge dans un contexte d'infection à VIH.
